# Individual response to transcranial direct current stimulation as a function of working memory capacity and electrode montage

**DOI:** 10.3389/fnhum.2023.1134632

**Published:** 2023-03-09

**Authors:** Inga Menze, Notger G. Mueller, Tino Zaehle, Marlen Schmicker

**Affiliations:** ^1^German Center for Neurodegenerative Diseases (DZNE), Magdeburg, Germany; ^2^Research Group Degenerative and Chronic Diseases, Movement, Faculty of Health Sciences Brandenburg, University of Potsdam, Potsdam, Germany; ^3^Department of Neurology, Otto-von-Guericke University, Magdeburg, Germany; ^4^Center for Behavioral Brain Sciences, Magdeburg, Germany

**Keywords:** tDCS, electrode montage, individual differences, working memory capacity, distractor inhibition, frontoparietal network

## Abstract

**Introduction:**

Attempts to improve cognitive abilities via transcranial direct current stimulation (tDCS) have led to ambiguous results, likely due to the method’s susceptibility to methodological and inter-individual factors. Conventional tDCS, i.e., using an active electrode over brain areas associated with the targeted cognitive function and a supposedly passive reference, neglects stimulation effects on entire neural networks.

**Methods:**

We investigated the advantage of frontoparietal network stimulation (right prefrontal anode, left posterior parietal cathode) against conventional and sham tDCS in modulating working memory (WM) capacity dependent transfer effects of a single-session distractor inhibition (DIIN) training. Since previous results did not clarify whether electrode montage drives this individual transfer, we here compared conventional to frontoparietal and sham tDCS and reanalyzed data of 124 young, healthy participants in a more robust way using linear mixed effect modeling.

**Results:**

The interaction of electrode montage and WM capacity resulted in systematic differences in transfer effects. While higher performance gains were observed with increasing WM capacity in the frontoparietal stimulation group, low WM capacity individuals benefited more in the sham condition. The conventional stimulation group showed subtle performance gains independent of WM capacity.

**Discussion:**

Our results confirm our previous findings of WM capacity dependent transfer effects on WM by a single-session DIIN training combined with tDCS and additionally highlight the pivotal role of the specific electrode montage. WM capacity dependent differences in frontoparietal network recruitment, especially regarding the parietal involvement, are assumed to underlie this observation.

## 1. Introduction

Transcranial direct current stimulation (tDCS) is a non-invasive neuromodulation method that has attracted immense attention in the context of cognitive research. Particularly, working memory (WM) has been a targeted cognitive function because of its relevance to other higher cognitive abilities (Engle and Kane, 2003; Johnson et al., 2013). Despite the abundance of tDCS research within this field, results still tend to be rather heterogeneous (Jantz et al., 2016). While improvements in WM performance–as resembled by shorter reaction times and higher accuracies–were frequently reported after anodal tDCS over frontal regions (Fregni et al., 2005; Andrews et al., 2011; Zaehle et al., 2011; Hoy et al., 2013; Lally et al., 2013), modulation by parietal tDCS led to more ambiguous results. As such, both anodal (Jones and Berryhill, 2012; Tseng et al., 2012; Hsu et al., 2014) and cathodal (Heimrath et al., 2012; Heinen et al., 2016) parietal stimulation were able to cause performance improvements in WM tasks. How can we explain the apparent paradox of same tDCS results despite different applications, or vice versa, different results despite the same application?

The variety of stimulation protocols–including, e.g., differing stimulation durations, current strengths or electrode montages–has often been criticized in tDCS research. In the present study, we aimed to investigate the impact of different electrode montages. The current majority of tDCS studies uses stimulation protocols with one electrode acting as the active electrode, placed over a specific brain area associated with cognitive functions which are intended to be modulated. The other electrode is considered the reference electrode and is placed over a region that is thought to be irrelevant to the task (Schmicker et al., 2011; Nasseri et al., 2015). These approaches, here referred to as conventional tDCS, tend to neglect the current flow between the active and the reference electrode (Shahid et al., 2014; Nasseri et al., 2015; Imburgio and Orr, 2018; Jaberzadeh et al., 2019). Depending on the relative position of the reference to the active electrode, current density under and between the two electrodes differs dramatically, resulting in possibly varying excitability of the targeted brain area or brain network (Faria et al., 2011; Nasseri et al., 2015; Foerster et al., 2018). This circumstance might contribute to ambiguous effects across tDCS studies. Regarding the aforementioned heterogenous findings in WM modulation by conventional parietal stimulation, improved performance after anodal tDCS was observed when the reference was placed over the contralateral cheek (Jones and Berryhill, 2012; Tseng et al., 2012; Hsu et al., 2014), while cathodal tDCS led to an increase in performance when the reference was placed over the contralateral brain region (Heimrath et al., 2012; Heinen et al., 2016).

Besides, electrode montage might also determine the modulation not only of a single brain area, but of an entire underlying brain network. In fact, tDCS was found to cause alterations in neuronal networks, manifesting, e.g., in increased resting-state (Keeser et al., 2011; Mancini et al., 2016; Li et al., 2019; Mencarelli et al., 2020) as well as modulated task-related functional network connectivity (Pisoni et al., 2018; Li et al., 2019; Jones et al., 2020; Sandrini et al., 2020). Hence, the question arises whether and how we can use tDCS to modulate brain networks and which electrode montage might induce an optimal network stimulation?

Focusing on WM, we first need to stress its close neuronal as well as behavioral association with selective attention (Gazzaley and Nobre, 2012). Both, the prioritization of target stimuli and the concurrent active suppression of irrelevant stimuli (distractor inhibition, DIIN), are thought to contribute to the capacity limit of WM (Awh and Vogel, 2008; Feldmann-Wüstefeld and Vogel, 2019; Liesefeld et al., 2020). While individuals with a high WM capacity (HCI) efficiently filter distractors, individuals with a low WM capacity (LCI) tend to strain their storage by an inefficient DIIN (Vogel and Machizawa, 2004; Vogel et al., 2005; McNab and Klingberg, 2008). Efficient DIIN in HCI might originate from robustness against the attentional capture of distractors and faster perceptual disengagement from them (Fukuda and Vogel, 2011), culminating in a decreased likelihood of mistakenly storing irrelevant items (Feldmann-Wüstefeld and Vogel, 2019). These specific DIIN abilities of LCI and HCI are further believed to be driven by the underlying frontoparietal network (Awh and Jonides, 2001; Constantinidis and Klingberg, 2016). Generally, DIIN and WM storage seem to involve nodes of this network to different extents. While DIIN has mainly been associated with activity of prefrontal areas (Gruber et al., 2006; McNab and Klingberg, 2008; Suzuki and Gottlieb, 2013; Ekman et al., 2016; Liesefeld et al., 2020), WM storage has more closely been linked to activity in the posterior parietal cortex (PPC) (Todd and Marois, 2004, 2005). Top down prefrontal control processes appear to additionally modulate activity in the PPC (Oliveri et al., 2001; Brass et al., 2005; Liesefeld et al., 2020). Moreover, higher frontoparietal network connectivity positively correlated with WM performance (Klingberg, 2006; Ekman et al., 2016; Assem et al., 2020) and training of WM resulted in increasing frontoparietal connectivity (Olesen et al., 2004; Klingberg, 2010; Takeuchi et al., 2010; Kundu et al., 2013; Constantinidis and Klingberg, 2016). These attributes of the frontoparietal network make it a potential research object for a network-oriented tDCS approach.

Some studies have already applied network-oriented tDCS successfully in this context. Stimulation of the frontoparietal network (unilateral frontal anode and parietal cathode) during a change detection task led to an improved WM performance in elderly participants (Arciniega et al., 2018). Moreover, improved processing speed after unilateral frontoparietal stimulation in an offline WM task correlated with stimulation related changes of the connectivity between the right middle frontal gyrus and the default mode network in young participants (Pupíková et al., 2021). Frontoparietal network-oriented tDCS could therefore be a successful application approach to evoke effects in the stimulated WM task.

Beyond that, due to the close relationship of both constructs, frontoparietal tDCS could also modulate previously described transfer effects of a DIIN training on WM (Schmicker et al., 2016; Li et al., 2017). Following this idea, we recently found that the degree of transfer effects by a combined single-session DIIN training with network-oriented tDCS onto an untrained WM change detection task depended on the initial WM capacity of participants (Schmicker et al., 2021). While HCI showed a positive transfer on WM performance under frontoparietal tDCS, LCI did not. We assumed that capacity dependent network characteristics were differently accentuated by this type of stimulation: While frontoparietal stimulation might have gated efficient network dynamics with an emphasis on frontally driven filtering in HCI, it might have disrupted important parietal compensational mechanisms in LCI. Yet, these earlier findings encompass different shortcomings. First, as we only compared sham to frontoparietal stimulation, tDCS effects cannot explicitly be ascribed to the network-oriented stimulation. For a clearer conclusion a comparison to another electrode montage is needed. Second, by using a median split inter-individual differences were only partly acknowledged.

Aiming to address these problems, we gathered subsequent data in the same experimental design as in Schmicker et al. (2021) using a conventional tDCS electrode montage with the anode placed over the right dorsolateral prefrontal cortex (DLPFC; F4) and an extracephalic cathode on the contralateral cheek. We used this data within a more robust reanalysis of our previous data (Schmicker et al., 2021). Hereby, we considered WM capacity as a continuous variable and analyzed data via linear mixed effect modeling to compare the conventional tDCS with already existing data of sham and frontoparietal stimulation. We aimed to draw more fine-grained conclusions regarding baseline WM capacity dependent tDCS effects. Based on our earlier findings (Schmicker et al., 2021), we hypothesized that conventional tDCS might enhance transfer independent from WM capacity, as it would solely modulate frontal filter related activity.

We focused on the comparison of transfer effects on WM by a single-session DIIN training under the different electrode montages. Our aim was to examine whether a network-oriented stimulation will yield individualized, WM capacity-related transfer effects as compared to sham and a conventional stimulation.

## 2. Materials and methods

### 2.1. Sample

Participants gave their written informed consent in accordance with the Declaration of Helsinki. They either received course credit or monetary compensation (10 €/h). The study was approved by the ethics committee of the University of Magdeburg (Germany). For this study we used an earlier sample of healthy participants between 18 and 30 years (Schmicker et al., 2021), which we expanded by a subsequent subsample of 47 participants receiving a conventional tDCS during a single-session DIIN training. Measurements took place from January to March 2020 and in October 2020 with an interruption due to COVID-19 pandemic related restrictions. The size of the subsequent sample was chosen to match the size of the previously studied samples. We recruited a few more individuals to avoid possible loss of data, e.g., due to outliers. Exclusion criteria for participation included neurological disorders, visual impairments, left-handedness, and metal implants in/on the head. The conventional stimulation group served as an active control group to the frontoparietal stimulation applied in our earlier work. Therefore, we (re)analyzed data from 133 participants (*n*_*sham*_ = 44, *n*_*conventional*_ = 47, *n*_*frontoparietal*_ = 42). All sample characteristics are reported in detail in the results section.

### 2.2. Experimental tasks and procedure

The experimental procedure and the conducted tasks were the same as described in detail in Schmicker et al. (2021) and are only briefly described here. Tasks were presented via Presentation^®^ (Version 20.1 12.04.17, Neurobehavioral Systems, Inc., Berkeley, CA, USA)^1^.

**FIGURE 1:**
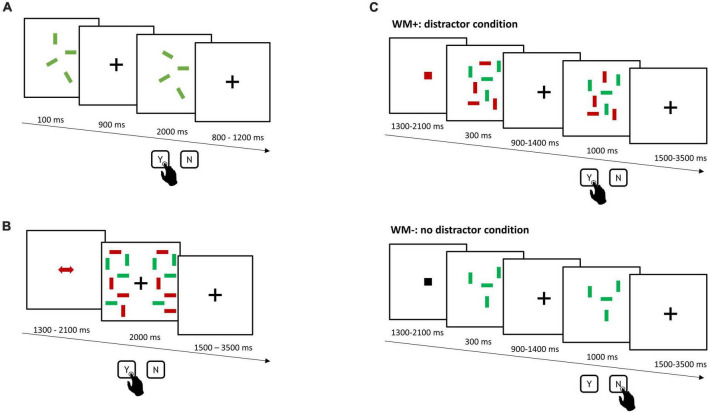
Schematic overview of the experimental tasks. **(A)** Independent assessment of WM capacity (*WMC task*). After a short encoding phase and delay, participants had to indicate, whether orientation of rectangles had changed. **(B)** Single-session training of distractor inhibition (*DIIN training*). Participants compared and reported orientation differences of color-cued target rectangles across the two test display halves. **(C)** Working memory transfer task (*WM*–*/WM*+). Participants encoded and remembered the orientation of color-cued target rectangles in the presence (top: distractor condition, WM+) or the absence of distractors (bottom: no distractor condition, WM–) and had to indicate, whether orientation of targets had changed. Stimulus size across all tasks was 1.43° × 0.29° viewing angle.

The experimental tasks comprised an independent assessment of WM capacity (*WMC task*, Figure 1A), using an adapted version of the change-detection task by Vogel et al. (2005). Participants had to encode and remember the orientation of green or red rectangles and report changes in their orientation after a short delay via key-press. Set-size ranged from 2 to 7 items. A change occurred in 50% of the trials (144 trials, 4 blocks).

In the single-session training of distractor inhibition (*DIIN training*, Figure 1B) participants had to compare the orientation of red or green rectangles across the two test display halves. Participants were asked to report on orientation differences. As red and green rectangles were presented together, a colored cue was presented before each trial to inform the color of target rectangles. The amount of target and distractor rectangles on each display halve ranged between 4 to 6 items each. The *DIIN training* was divided into 6 experimental blocks with 50 trials each.

Finally, the primary outcome measure was a change-detection task (*WM−/WM*+, Figure 1C), which comprised a condition without and another condition with distractors. Participants encoded and remembered the orientation of either red or green target rectangles in the absence (WM−) or the presence of distractors (WM+) and reported changes therein after a short delay. A cue before each trial informed participants of the color of target rectangles, whereby a black cue indicated an upcoming WM− trial. The amount of target rectangles ranged from 4 to 6 items, with the same amount of distractor rectangles in WM+. A change occurred in 50% of the trials (144 trials, 4 blocks).

Participants first underwent the assessment of the WMC task (10 min). The pre assessment of WM−/WM+ followed (30 min). Participants were then connected to tDCS and underwent the single-session DIIN training (45 min) during which they received sham (first 30 s) or verum stimulation (first 10 min). Before they conducted the final post assessment of the WM−/WM+ task (30 min), participants filled out a questionnaire, reporting on experienced adverse effects during or after the stimulation (tingling/itching sensation, pain/burning sensation, headache, nausea) on a 5-point Likert scale.

### 2.3. tDCS protocol

Three stimulation protocols were used for this study: a sham stimulation, lasting 30 s and two verum stimulation protocols, lasting 10 min. We opted for a stimulation duration of 10 min to avoid possible sensory or motoric after effects (Ohn et al., 2008) that might have confounded performance in the subsequent transfer task.

**FIGURE 2:**
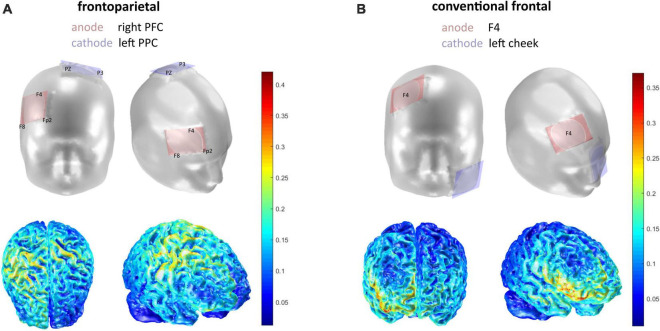
Electrode montages in frontal and lateral view. **(A)** Frontoparietal stimulation application. **(B)** Conventional stimulation application. The current density is depicted in Joule beneath (see colorbar for scale). Simulation of electrode montage and current density was executed with COMETS 2.0.

The frontoparietal verum stimulation with a right prefrontal anode and a left posterior parietal cathode as well as the sham stimulation are described in Schmicker et al. (2021). The aim of the frontoparietal stimulation set-up was to target the middle frontal gyrus via the anode, while targeting the left intraparietal sulcus via the cathode. Using COMETS 2.0 (Matlab R2018a, The MathWorks, Inc., Natick, MA, United States; Lee et al., 2017), the electrode positions depicted in Figure 2A were chosen. According to the international 10–20 system for electroencephalography (EEG), the anode was hence placed between Fp2, F4, and F8, and the cathode between PZ and P3.

For the conventional protocol, we used a tDCS set up with the anode over the right prefrontal cortex (F4 according to international 10–20 system) and the cathode placed over the contralateral cheek (extracephalic reference; Figure 2B). tDCS was applied via the DC-Stimulator by neuroConn, using sponge-electrodes (5 × 7 cm) that were soaked in 0.9% saline solution. Cephalic electrodes were positioned and fastened using EEG-caps. The extracephalic electrode in the conventional stimulation was secured on the contralateral cheek using hypoallergenic tape. The current strength in all protocols was set to 1.5 mA with a fade-in and fade-out interval of 1 s. The stimulation was delivered within the first 10 min of the single-session DIIN training, i.e., in the first two experimental blocks. Stimulation was single-blinded to the subjects and examiners were following a standardized protocol in order to avoid any bias.

### 2.4. Data analyses

We used a mixed design, with the within-subject factor time resulting from the six experimental blocks in the single-session DIIN training and the pre-post measurements of WM−/WM+, respectively. The between-subject factors included stimulation (frontoparietal vs. sham vs. conventional) and WM capacity. We calculated the individuals’ WM capacity according to Pashler (1988), as the WMC paradigm was designed as a whole display retrieval (Rouder et al., 2011). Our main variables of interest were accuracy (% correct) and RT in the transfer WM−/WM+ task. Of secondary interest was accuracy (% correct) in the trained single-session DIIN task to assess training effects.

Outliers of the initial sample of *n* = 133 were identified as participants whose performance in the WM−/WM+ paradigm as well as in the single-session DIIN training deviated ± 2 *SD* from the mean accuracy level. Furthermore, participants whose WM capacity was above Q3 + 1.5 × IQR or below Q1–1.5 × IQR from the median of the sample were identified as outliers and were excluded from analysis. In sum, nine participants were excluded (*n*_sham_ = 1, *n*_frontoparietal_ = 3, *n*_conventional_ = 5) leaving an analyzable sample of *n* = 124 (*n*_sham_ = 43, *n*_frontoparietal_ = 39, *n*_conventional_ = 42; please note that due to new analysis whole-sample distributions may slightly deviate from our previous work in Schmicker et al., 2021). We checked upon equivalence across the three stimulation groups in the main variables of interest, i.e., accuracy (% correct) and RT in the WM−/WM+ task as well as baseline WM capacity via univariate ANOVAs. Furthermore, we evaluated comparability of age, distribution of sex, assessment of having received verum stimulation, and reported adverse effects of stimulation across groups.

We used linear mixed effects (LME) modeling to elaborate whether frontoparietal stimulation was yielding differential effects depending on WM capacity as compared to conventional and sham stimulation. We hence used the frontoparietal stimulation as the reference stimulation group of the fixed factor stimulation. We checked preconditions of linearity, homoscedasticity, and normality of residuals by visually inspecting residual plots and Q-Q plots. Influential data points were examined via Cook’s distance, with a cut-off value suggested by Van der Meer et al. (2010) defined as four divided by the number of groups. However, no influential data points had to be excluded. Effect size estimates using beta coefficients, 95% confidence intervals and an alpha level of *p* < 0.05 are reported.

We initiated our analysis with examining performance changes in the single-session DIIN training. We compared a null model including the fixed factors experimental block (1–6; here “time”), stimulation (frontoparietal vs. sham and conventional stimulation) and their interaction to a full model further including WM capacity in the interaction term. Both models were compared via a likelihood ratio test to evaluate if WM capacity contributed significantly to the performance progression in the different stimulation groups. A correlated random slope and a random intercept per subject were integrated to acknowledge individual differences in all models. Superiority of the random intercept and random slope model over a random intercept only model was tested via likelihood ratio test.

Our main research question focused on possible transfer effects from the single-session DIIN training on the WM−/WM+ task, particularly its sub conditions. First, we ran correlational analyses of the extracted random slope of DIIN training and participants’ individual performance increase in WM−/WM+ (Δ performance = performance _post_−performance _pre_). Pearson correlations were used if normal distribution was given; in case of non-normally distributed data, Kendall’s correlation was used. Second, to assess WM capacity dependent effects, we ran correlational analyses between WM capacity and Δ performance as well as reaction time (RT) differences (Δ RT = RT _post_−RT _pre_) in WM−/WM+ across stimulation groups. Third, we tested accuracy and RT as a function of the fixed factors time (pre to post) and stimulation (frontoparietal vs. sham and conventional stimulation) via LME modeling. We used log-transformed RTs to address assumptions of linearity and normal distribution. We compared this model to a model including WM capacity and its interactions with time and stimulation as fixed factors by means of a likelihood ratio test, to evaluate the impact of WM capacity on changes in accuracy and RT. Random intercepts per subjects in all models accounted for individual differences. Models with the same fixed and random effect structure were separately calculated for the performance in no-distractor and distractor conditions, respectively, to evaluate if effects were specific for a certain condition. Results on the overall performance are reported in the Supplementary material.

Data analysis was conducted in R version 4.0.2 using RStudio version 1.3.1073 (R Core Team, 2020). LME modeling was conducted via the packages lme4 (Bates et al., 2015), lmerTest (Kuznetsova et al., 2017), influence.ME (Nieuwenhuis et al., 2012), and psych (Revelle, 2020). Figures were created with the packages ggplot2 (Wickham, 2016) and sjplot (Lüdecke, 2018).

## 3. Results

### 3.1. Sample characteristics

Univariate ANOVAs showed that baseline performance in WM−/WM+ as well as in WM capacity did not differ between stimulation groups (Table 1). A Kruskal–Wallis test showed differences in reports of adverse effects of stimulation in terms of tingling/itching [χ^2^(2) = 22.48, *p* < 0.001], which was more frequently reported by subjects of the frontoparietal (*p* = 0.002) and conventional (*p* < 0.001) stimulation group as compared to sham. The report on other adverse effects did not significantly differ between stimulation groups. We still considered blinding successful, as there were no significant differences between groups regarding their perception of having been stimulated.

**TABLE 1:** Sample characteristics of the three stimulation groups.

	Sham (*n* = 43)	Frontoparietal (*n* = 39)	Conventional (*n* = 42)	
% Females	65.11	64.10	69.05	*p* = 0.882
% Thought to be stimulated	72.09	74.36	88.10	*p* = 0.158
Tingling/itching	2.81 ± 1.12	3.64 ± 1.09	4.00 ± 1.01	*p* < 0.001
Pain/burning	1.88 ± 1.07	1.90 ± 0.99	2.17 ± 1.21	*p* = 0.503
Headache	1.72 ± 0.85	1.41 ± 0.72	1.36 ± 0.62	*p* = 0.051
Nausea	1.16 ± 0.43	1.21 ± 0.52	1.19 ± 0.51	*p* = 0.974
Age	23.37 ± 2.78	23.41 ± 2.55	22.83 ± 2.32	*p* = 0.405
Accuracy WM−/WM+_pre_	76.01 ± 5.50	76.83 ± 6.11	77.30 ± 6.38	*p* = 0.353
RT in ms WM−/WM+_pre_	1,030.62 ± 242.75	973.58 ± 228.71	954.21 ± 222.40	*p* = 0.129
WM capacity_pre_	2.21 ± 0.59	2.37 ± 0.47	2.25 ± 0.57	*p* = 0.680

Mean ± standard deviation.

### 3.2. Effects within the single-session DIIN training

**FIGURE 3:**
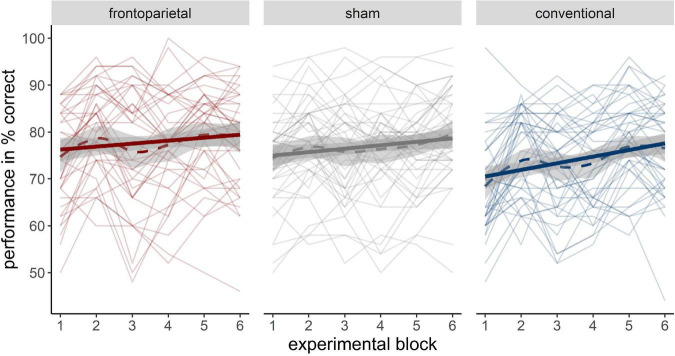
Performance increase across different stimulation groups (**left**: frontoparietal; **middle**: sham; **right**: conventional). Bold solid trajectories depict the group mean within a 95% CI. Thin solid lines resemble individual performance trajectories of participants. Bold dashed lines show the loess smoothed group trajectory.

The descriptive training performance in all three groups can be retrieved from Supplementary Table 1. In the LME analysis, the random slope and intercept model, was superior to a random intercept only model [χ^2^(2) = 18.86, *p* < 0.001]. The model including the fixed factors time and stimulation revealed a positive performance increment across experimental blocks (Figure 3). Besides, it showed that the performance increase tended to be greater in participants who received conventional stimulation than in participants who received frontoparietal stimulation (time × conventional: *β* = 0.78, *SE* = 0.42, *95%-CI* [−0.04; 1.60]), although the frontoparietal group outperformed the conventional group in general (conventional: *β* = −6.53, *SE* = 2.23, *95%-CI* [−10.94; −2.11]). Performance patterns between sham and frontoparietal stimulation did not differ substantially. The model parameters of the random slope model can be retrieved from Supplementary Table 2. Adding WM capacity as another fixed factor did not increase model likelihood [χ^2^(6) = 8.33, *p* = 0.215].

### 3.3. Working memory capacity dependent transfer effects on working memory accuracy

An overview of the accuracy and reaction times in the WM−/WM+ paradigm and its respective sub conditions across the stimulation groups can be retrieved from Supplementary Table 3.

First, we extracted the random slope of participants in the single-session DIIN training and correlated it with their individual performance difference in the overall, the no distractor and the distractor condition of WM−/WM+, respectively, across stimulation groups. However, gains in the single-session DIIN training only correlated weakly to moderately with increments in WM−/WM+ or its sub conditions (Table 2).

**TABLE 2:** Correlations between random slopes within distractor inhibition (DIIN) training across stimulation groups with pre-post performance difference in WM**−**/WM+.

	ΔWM−/WM+_overall_	ΔWM−	ΔWM+
Slope _sham_	0.230	0.100^K^	0.150
Slope _frontoparietal_	0.075	0.012^K^	0.081
Slope _conventional_	−0.047	0.051^K^	−0.062

Correlations flagged with ^K^ were run with Kendall’s method due to non-normally distributed data. All other correlations were run via Pearson’s method.

Second, grouped correlations between WM capacity and Δ performance in the overall, the no distractor and the distractor condition, respectively, were conducted. In the overall condition, a trend for a positive correlation between WM capacity and Δ performance_overall_ showed in the frontoparietal stimulation group (*r* = 0.35, *p* = 0.027, *p*_*corr*_ = 0.082), but neither in the sham (*r* = −0.20, *p* = 0.200, *p_*corr*_* = 0.60) nor in the conventional stimulation group (*r* = −0.04, *p* = 0.817, *p_*corr*_* = 1.0). In the no distractor condition no significant correlations between WM capacity and Δ performance_no distractor_ for either group could be shown. In the distractor condition, we found a tendency for a positive correlation between Δ performance_distractor_ and WM capacity in the frontoparietal stimulation group (*r* = 0.30, *p* = 0.066, *p_*corr*_* = 0.198) as opposed to a small to moderate negative trend in the sham stimulation group (*r* = −0.27, *p* = 0.078, *p_*corr*_* = 0.235). In the conventional group no such relation was observed (*r* = −0.06, *p* = 0.688, *p_*corr*_* = 1.0).

Third, we used LME modeling to elaborate on the relations of performance changes and WM capacity in more detail. Across the sub conditions WM− and WM+ of the task, the full models including WM capacity as fixed factor were superior to the respective null model only including time and stimulation [no distractor condition: χ^2^(6) = 15.23, *p* = 0.019; distractor condition: χ^2^(6) = 25.05, *p* < 0.01; overall WM−/WM+ performance: χ^2^(6) = 25.12, *p* < 0.01]. Accordingly, only the full models will be addressed in the following. The respective results of the null models can be retrieved from the Supplementary Tables 4–6. Based on our earlier findings showing distinct transfer patterns after stimulation in sub conditions of WM−/WM+, we here focus on WM− and WM+, respectively, and report the full model for the overall performance only in Supplementary Table 7. Please note, that we also provide LME models for all conditions of WM−/WM+ with sham as reference stimulation group in Supplementary Table 9 in order to allow for the direct comparison of sham and conventional stimulation.

#### 3.3.1. Performance in distractor condition

**FIGURE 4:**
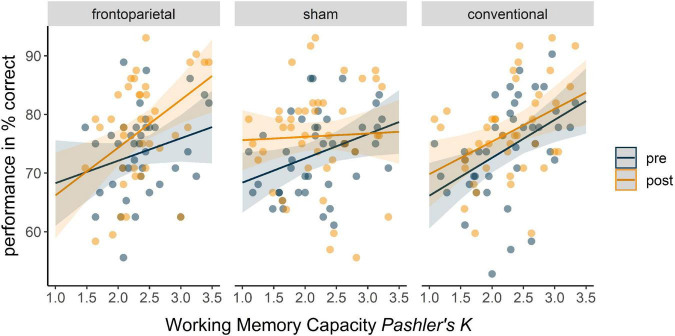
Predicted overall performance in distractor condition (WM+) of the WM–/WM+ change-detection task in the three stimulation groups in dependence of WM capacity. Fixed factors of the full model included time, stimulation, WM capacity, and their interaction. Blue trajectories depict performance in the pre assessment of WM+, orange trajectories depict performance in the post assessment of WM+ across the WM capacity continuum. Dots represent participants’ performance in the pre (blue) and post (orange) assessment.

In the distractor condition, we observed a significant negative three-way interaction of time × sham × WM capacity, indicating stronger performance increases from pre to post with increasing WM capacity in frontoparietal stimulation as compared to sham (*β* = −7.88, *SE* = 3.15, *95%-CI* [−14.10; −1.67]). For the frontoparietal stimulation performance differences from pre to post tended to accrete with increasing WM capacity (Figure 4), represented by a trend in time × WM capacity interaction (*β* = 4.32, *SE* = 2.51, *95%-CI* [−0.64; 9.27]). However, if WM capacity was low, sham stimulation seemed more profitable (time × sham: *β* = 17.20, *SE* = 7.44, *95%-CI* [2.50; 31.91]).

We observed similar differential tendencies for the conventional stimulation compared to frontoparietal stimulation, which remained, however, insignificant (time × WM capacity × conventional: *β* = −5.22, *SE* = 3.22, *95%-CI* [−11.58; 1.14]; time × conventional: *β* = 10.97, *SE* = 7.66, *95%-CI* [−4.15; 26.10]). All indices of the full model can be retrieved from Table 3.

**TABLE 3:** Full linear mixed model for the distractor condition WM+.

	β	Std. error	95%-CI		
Intercept	64.45	6.22	52.20	76.71	***
Time	-6.40	6.06	-18.36	5.56	
WMC	3.82	2.58	-1.25	8.90	
Sham	-0.22	7.65	-15.28	14.84	
Conventional	-4.76	7.87	-20.26	10.73	
Time × WMC	4.32	2.51	-0.64	9.27	
Time × sham	17.20	7.44	2.50	31.91	*
Time × conventional	10.97	7.66	-4.15	26.10	
WMC × sham	0.31	3.23	-6.05	6.68	
WMC × conventional	2.64	3.31	-3.88	9.15	
Time × WMC ×sham	-7.88	3.15	-14.10	-1.67	*
Time × WMC × conventional	-5.22	3.22	-11.58	1.14	

Model: performance∼time × stimulation × WM capacity + (1| participant). WMC = WM capacity. Random intercept SD = 5.45.

***p < 0.001; *p < 0.05; *p* < 0.1.

#### 3.3.2. Performance in no distractor condition

In the no distractor condition, the effects follow the same pattern as in the distractor condition, however, they remained insignificant (Supplementary Table 8).

### 3.4. Transfer effects on reaction times in working memory task

We first ran grouped correlational analyses for RT differences (Δ RT) between pre and post with WM capacity. No significant correlations were found between ΔRT in the overall, the no distractor or the distractor condition (Supplementary Table 10). For the LME analyses we therefore compared a null model only including time as a fixed factor against a full model extending the model by a time × stimulation interaction.

**FIGURE 5:**
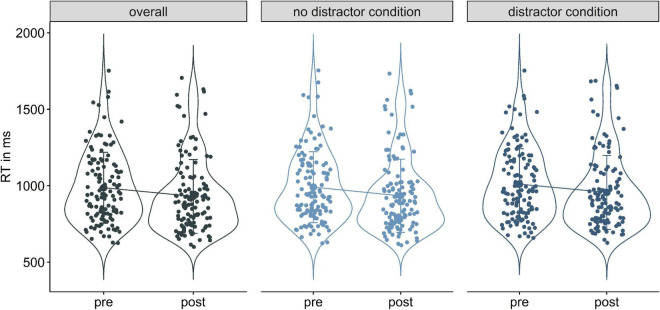
Violin plots of reaction time decreases from pre to post in the overall **(left)**, no distractor condition **(middle)**, and distractor condition **(right)**.

In all sub conditions, full models including stimulation were not more informative than the null models [overall condition: χ^2^(4) = 2.59, *p* = 0.63; no distractor: χ^2^(4) = 4.54, *p* = 0.34; distractor condition χ^2^(4) = 2.15, *p* = 0.71]. Stimulation hence did not affect RT in either condition of WM−/WM+. Yet, across all conditions time had a significant impact on RT, implying faster reaction times from pre to post assessment (*β*_*overall*_ = −0.06, *SE*_*overall*_ = 0.01, *95%-CI* [−0.09; −0.04]; *β*_*nodis*_ = −0.07, *SE_*nodis*_* = 0.01, *95%-CI* [−0.09; −0.04]; *β*_*dis*_ = −0.06, *SE_*dis*_* = 0.01, *95%-CI* [−0.09; −0.03], Figure 5 and Supplementary Table 11). LME models on untransformed RTs showed the same pattern of effects (Supplementary Table 11).

## 4. Discussion

This study assessed the modulation of transfer effects from a single-session DIIN training on WM performance by different tDCS electrode montages. Although single-session DIIN training performance progression did not correlate with the performance gains in the transfer WM task, we showed WM capacity dependent effects for the different tDCS electrode montages. We observed accentuations of performance gains after frontoparietal and sham stimulation, respectively, which were especially striking in the distractor condition. Whereas the frontoparietal stimulation seemed to have a positive impact on performance changes the higher the WM capacity, sham stimulation, i.e., a sole training of DIIN, was rather beneficial the lower the WM capacity. By contrast, the conventional stimulation did not seem to elicit WM capacity specific accentuations of performance gains in the WM transfer, but instead general, subtle performance increases.

### 4.1. Single-session distractor inhibition training

All groups exhibited a performance increment across experimental blocks in the single-session DIIN training. There was a trend for a stronger increase in the conventional stimulation group as compared to the frontoparietal stimulation group, which remained, however, insignificant. As the conventional stimulation group showed a lower performance in the DIIN training in the first experimental block to begin with, this trend might simply indicate greater “room for improvement.” Yet, the stimulation might have indeed had a positive effect on training improvement. By predominantly targeting prefrontal areas, the conventional stimulation might have modulated associated processes of top-down control over task-specific posterior areas and influenced response-stimulus associations (Brass et al., 2005; Anderson et al., 2007; Bressler et al., 2008; Morishima et al., 2009; Harding et al., 2015). An interaction of both possibilities–in terms of greater potential for improvement being rather exploited under conventional stimulation–could be possible as well.

We note, however, that effects of WM capacity as well as stimulation on performance within the single-session DIIN training were generally sparse. We assume that possible neuromodulatory effects were annihilated by the long task duration of ca. 45 min, so that strong training effects might have concealed possible weaker stimulation effects. Filmer et al. (2017) showed comparable training improvements across different stimulation groups in a 4-day multitasking training, too. Like here, their combination of training with anodal stimulation of the left DLPFC still elicited near transfer effects to a visual search task and to an untrained multitasking paradigm. Other studies showed near transfer effects after training combined with tDCS, too, especially in longitudinal designs (Richmond et al., 2014; Trumbo et al., 2016; Weller et al., 2020). Hence, stimulation effects on our applied training might have only become apparent with a more extensive training regime (Weller et al., 2020). Additionally, we note that there might be other inter-individual factors, such as age or education, that could exceed stronger influence on performance patterns beyond stimulation or WM capacity in the DIIN training which, however, could not be reliably investigated due to the homogenous sample.

Although the training increment in the current study did not correlate with the performance gains in the primary WM outcome task, different trajectories in the transfer task were shown in dependence of WM capacity and stimulation. Hence a more indirect influence might have originated from the stimulation that led to the specific transfer effects on WM. As such, we assume a preparatory entrainment of neuronal processes via the combination of stimulation and single-session DIIN training which eventually enabled greater performance in the transfer WM task but not in the online stimulated task. It was shown before, that online tDCS induced effects on the physiological level in the absence of behavioral effects (Hill et al., 2018).

### 4.2. Capacity and stimulation dependent transfer effects on working memory

Correlational as well as LME analysis showed that effects on the transfer WM task performance depended on the interaction of stimulation and baseline WM capacity. This phenomenon seems to especially occur in distractor conditions. As described in our earlier work (Schmicker et al., 2021), the higher the initial WM capacity, the stronger the performance increased in the transfer WM task after frontoparietal stimulation. In contrast, sham stimulation, i.e., sole DIIN training, was rather beneficial when the initial WM capacity is lower. Additionally, we now show that the conventional stimulation led to effects that were independent of initial WM capacity. Across the WM capacity spectrum, participants showed comparable, yet subtle, performance gains from pre to post that did not differ significantly from those of the frontoparietal stimulation group. In comparison to performance after frontoparietal stimulation, however, HCI receiving conventional stimulation did not yield the “extraordinary” performance gain. Similarly, LCI did not reach the same performance gain after conventional as compared to sham stimulation. Therefore, while sham and frontoparietal stimulation seemed to better accentuated outcomes based on initial WM capacity, conventional stimulation caused results that were somewhat in between (see also Supplementary Table 9). As mentioned, these characteristic trajectories were especially apparent in the distractor condition. In the no distractor condition electrode montage and WM capacity did not interact, resulting in comparable performance progression of the three stimulation groups. Moreover, these effects were only present in terms of accuracy, but did not survive in RT analyses. What could be the reason to this restriction of effects to the distractor condition?

Absent differences between conventional and frontoparietal stimulation indicated that the network-oriented stimulation is not necessarily superior to the conventional stimulation. Yet, our findings suggest, that different electrode montages might accentuate stimulation effects in dependence of initial WM capacity. It has been argued that effects of tDCS vary with the initial recruitment and activity of the stimulated brain areas and that baseline performance could be representative of such underlying neuronal states (Silvanto et al., 2008; Ziemann and Siebner, 2015; Hsu et al., 2016; Dubreuil-Vall et al., 2019; Emonson et al., 2019; Esposito et al., 2022). As stimulation protocols (Teo et al., 2011; Moos et al., 2012; Filmer et al., 2017), as well as baseline performance (Benwell et al., 2015; Learmonth et al., 2015; Splittgerber et al., 2020) influence stimulation effects, they should hence be considered in the analysis and interpretation.

As mentioned before, selective attention and WM go hand in hand on a behavioral as well as neuronal level (Vogel and Machizawa, 2004; Vogel et al., 2005; Cowan and Morey, 2006; Mayer et al., 2007; McNab and Klingberg, 2008; McCabe et al., 2010; Schmicker et al., 2016). Since the connectivity within the frontostriatal pathway was found to correlate positively with WM capacity and DIIN (Klingberg, 2006; McNab et al., 2008; Baier et al., 2010; Nee and Brown, 2013; Darki and Klingberg, 2015; Ekman et al., 2016), it could be assumed, that the frontoparietal stimulation applied here might have triggered this pathway optimally and enhanced frontal filtering in HCI even more (Schmicker et al., 2021). However, in individuals with lower WM capacity, the opposite seemed to be the case, resulting in lower performance gains after frontoparietal stimulation during single-session DIIN training as compared to sham. In contrast, after conventional stimulation all participants showed performance increase regardless of their WM capacity. Based on these findings, we hypothesize, that the parietal involvement is key to explaining the interaction of electrode montage and WM capacity. Firstly, individuals with a lower WM capacity appear to encode all presented stimuli, instead of selecting relevant information early in the encoding phase, thereby straining capacity resources (Vogel and Machizawa, 2004; Vogel et al., 2005; Linke et al., 2011). Secondly, besides its association with WM storage, the PPC also seems to be associated with attention related processes and hence seems to be involved in the interplay of WM and DIIN (Corbetta and Shulman, 2002; Corbetta et al., 2008; Berryhill et al., 2011; Moos et al., 2012). Thereby, an assumed role of the PPC was to maintain attention on WM representations, preventing them from decaying (Berryhill, 2012)–a process which was found to be interrupted by cathodal stimulation (Berryhill et al., 2010). Taken together, we assume that individuals with lower WM capacity rely more strongly on this parietal involvement for successful change detection WM performance, a pathway that might have been interrupted by the cathodal stimulation in frontoparietal tDCS. Contrasting to that, individuals with higher WM capacity might be able to successfully engage a different pathway to manage such tasks, thereby avoiding disruptions by the cathodal stimulation. Interestingly, although individuals with lower WM capacity also profited from conventional stimulation in distractor conditions, transfer effects were still stronger in the sham condition for these individuals. It might be, that although conventional stimulation primarily targeted modulation of prefrontal related filtering, it still introduced noise into supposedly putative sensitive network dynamics.

## 5. Conclusion and limitations

All in all, an exact understanding of the frontoparietal network processes and possible fine-tuned feedback relations of involved brain areas, their vulnerability to tDCS modulation and their role in transfer in the context of DIIN and WM still remains elusive. Therefore, examining other electrode montages in this transfer context would be of help to further clarify the roles of frontal and parietal regions in DIIN, e.g., by using reversed polarity electrode montages than those described here.

Moreover, functional activity and connectivity in targeted brain regions and networks–in this case the frontoparietal and frontostriatal connections–could be a predictor for both behavioral performance and respondence to neuromodulation. As the current study and related assumptions were made upon behavioral data, we strongly encourage carefully planned large-scale multimodal studies combining tDCS with behavioral tasks as well as neuroimaging and/or neurophysiological data (Woods et al., 2016; Wörsching et al., 2016; Esmaeilpour et al., 2020), to draw stronger conclusions. In this context, we also emphasize, that apart from neuronal polarization and neuroplasticity, other influential factors might have influenced our results. As such, further proposed transient mechanisms of action of tDCS include modulation of neurotransmitter transmission (Stagg et al., 2009; Hunter et al., 2015; Fonteneau et al., 2018), modulation of peripheral nerves that indirectly affect transmitter pathways (Vanneste et al., 2020; van Boekholdt et al., 2021), increasing cerebral perfusion (Wachter et al., 2011; Stagg et al., 2013), and affecting cerebral (micro)vasculature by increasing blood-brain barrier permeability (Shin et al., 2020; Xia et al., 2020). The unique contribution and interplay of these putative influential factors are still to be elucidated–especially in non-motor brain areas (Stagg et al., 2018).

Carefully assessed large-scale studies are also needed to circumvent possible inflation of effects due to publication bias or small samples, stressing randomly strong effects (Minarik et al., 2016; Medina and Cason, 2017; Filmer et al., 2020). Considering the three stimulation groups and concurrent consideration of inter-individual variability in WM capacity, the sample of the current study remains rather small. Additionally, we note that generalizations to other cohorts are limited, since we here only studied healthy young subjects. Therefore, further studies are required to examine the stability of these effects in other cohorts. Besides, data of the conventional stimulation group was subsequently assessed, which could have caused systematic variability across stimulation groups, e.g., due to changes in the lab, seasonal effects on cognitive performance (Meyer et al., 2016) or effects due to the ongoing COVID-19 pandemic and related restrictions (Menze et al., 2022). We assured, however, that examiners followed a standard operating procedure, and that measurements were conducted in the same facilities and rooms as previously. Moreover, measurements took place in autumn/winter in both the previous and the subsequent sample. Finally, we consider the likelihood of such systematic biases as low since the stimulation groups did not differ significantly in their performance in the main variables of interest before stimulation.

Lastly, though meta-analyses have criticized the huge variety of stimulation protocols, this critique seems to address a symptom rather than a cause of failed attempts to replicate or show strong tDCS effects (Horvath et al., 2015; Medina and Cason, 2017; Imburgio and Orr, 2018; Filmer et al., 2020; de Boer et al., 2021). Like previous studies, our data corroborates the notion that there is no such thing as a “one size fits all” stimulation, but instead supports the assumption that optimal stimulation results can only be reached if stimulation protocols are carefully adapted in accordance with individual factors that might predict respondence to tDCS. Identifying individual characteristics and circumstances for respondence to tDCS will be critical (Krause and Cohen Kadosh, 2014; Ziemann and Siebner, 2015; Liu et al., 2018; Luque-Casado et al., 2019, 2020), since personalized stimulation application seems to become a promising approach to assure effectiveness of tDCS (Antonenko et al., 2019; Indahlastari et al., 2021; see also Woods et al., 2016; Esmaeilpour et al., 2020). We acknowledge, however, that our study is limited regarding generalization to other stimulation parameters, since we here focused on the interaction of inter-individual differences in WM capacity and electrode montage in the context single-session cognitive training in a young and healthy sample. We therefore also encourage further investigation and the effort of large-scale studies to examine the interplay of different stimulation parameters, e.g., stimulation duration or current strength, and their interplay with inter-individual factors.

A deeper understanding of tDCS effects on the individual level is ultimately required. More studies directly investigating effects of different electrode montages–and/or other stimulation parameters–in dependence on individual factors are needed (Splittgerber et al., 2020). Identifying strong proxies which indicate underlying individual neuronal processes and response to non-invasive brain stimulation can therefore be helpful. Our data suggests that baseline WM capacity might be one such indicator for neuronal processes that could be influenced inter-individually via tDCS.

## Data availability statement

The raw data supporting the conclusions of this article will be made available upon reasonable request.

## Ethics statement

The studies involving human participants were reviewed and approved by the Ethics Committee of the University of Magdeburg (Germany). The patients/participants provided their written informed consent to participate in this study.

## Author contributions

MS and IM developed the idea for this experiment and designed the tasks. IM collected and analyzed the data and drafted the manuscript. IM, MS, TZ, and NM contributed to the discussion of content-related issues and to the critical revision of the manuscript, and approved the submitted version.

## Footnotes

1
http://www.neurobs.com
