# Supplementary material for: Individual response to transcranial direct current stimulation as a function of working memory capacity and electrode montage
Source: Front Hum Neurosci. 2023 Mar 9;17:1134632. doi: 10.3389/fnhum.2023.1134632 (PMC10034341; doi:10.3389/fnhum.2023.1134632)
Supplement: Supplementary file 1 [file Data_Sheet_1.docx]

Supplementary Material

**Individual response to transcranial direct current stimulation as a function of working memory capacity and electrode montage**

**Menze, Inga^*^, Mueller, Notger G., Zaehle, Tino, Schmicker, Marlen**

*** Correspondence:** Inga Menze: [inga.menze@dzne.de](mailto:inga.menze@dzne.de)

# Supplementary Tables

Table 1. Descriptive statistics for the single-session DIIN training across groups.

|  |  | **sham (*n*=43)** | **frontoparietal (*n*=39)** | **conventional (*n*=42)** |
| --- | --- | --- | --- | --- |
| **overall** | accuracy in % correct | 76.83 ± 8.69 | 77.84 ± 9.40 | 74.04 ± 7.89 |
|  | RT in ms | 2025.33 ± 422.53 | 2016.69 ± 374.33 | 1896.04 ± 359.98 |
|  |  |  |  |  |
| **experimental blocks** | accuracy block 1 | 74.13 ± 9.73 | 74.05 ± 10.33 | 67.90 ± 9.88 |
|  | accuracy block 2 | 77.91 ± 9.78 | 80.97 ± 9.24 | 75.81 ± 9.03 |
|  | accuracy block 3 | 75.77 ± 10.66 | 75.90 ± 13.15 | 72.67 ± 10.68 |
|  | accuracy block 4 | 76.37 ± 11.05 | 77.23 ± 11.17 | 73.38 ± 9.68 |
|  | accuracy block 5 | 77.02 ± 9.80 | 80.15 ± 10.18 | 78.33 ± 9.52 |
|  | accuracy block 6 | 79.67 ± 9.91 | 78.72 ± 11.09 | 76.14 ± 10.13 |

*Annotations.* Mean ± standard deviation***.***

Table 2. Random slope and random intercept model for single-session DIIN training.

|  | **β** | **Std. Error** | | | **95%-CI** | |  |
| --- | --- | --- | --- | --- | --- | --- | --- |
| **intercept** | 75.62 | | 1.61 | 72.44 | | 78.80 | *** |
| **time** | 0.63 | | 0.30 | 0.042 | | 1.23 | * |
| **sham** | -1.37 | | 2.22 | -5.76 | | 3.02 |  |
| **conventional** | -6.53 | | 2.23 | -10.94 | | -2.11 | ** |
| **time x sham** | 0.10 | | 0.41 | -0.72 | | 0.92 |  |
| **time x conventional** | 0.78 | | 0.42 | -0.04 | | 1.60 | . |

*Annotations.* Model: performance ~ time x stimulation + (1+time|participant). Random intercept SD = 8.38. Random slope SD = 1.22. ***p < .001; **p < .01; *p < .05; p < .1

Table 3. Descriptive statistics across the stimulation groups for the WM-/WM+ paradigm and it sub conditions.

|  |  | **sham (*n*=43)** | **frontoparietal (*n*=39)** | **conventional (*n*=42)** |
| --- | --- | --- | --- | --- |
| **overall** | pre | 76.10 ± 5.50 1030.62 ± 242.75 | 76.83 ± 6.11 973.58 ± 228.71 | 77.30 ± 6.38 954.21 ± 222.40 |
|  | post | 79.59 ± 7.39 968.49 ± 289.16 | 80.77 ± 6.86 913.36 ± 216.77 | 80.69 ± 6.78 907.31 ±208.73 |
|  |  |  |  |  |
| **WM-** | pre | 78.82 ± 6.73 1009.43 ± 249.30 | 80.16 ± 6.58 945.96 ± 227.39 | 80.33 ± 6.06 924.90 ± 216.03 |
|  | post | 82.91 ± 7.28 942.95 ± 216.03 | 84.19 ± 6.36 888.61 ± 217.41 | 84.59 ± 7.39 877.54 ± 204.52 |
|  |  |  |  |  |
| **WM+** | pre | 73.35 ± 6.76 1052.26 ± 243.58 | 73.50 ± 7.43 1001.17 ± 234.39 | 74.25 ± 8.33 983.17 ± 231.98 |
|  | post | 76.29 ± 8.85 994.08 ± 292.73 | 77.32 ± 9.02 938.42 ± 222.74 | 76.79 ± 8.04 937.28 ± 218.54 |

*Annotations.* Mean ± standard deviation is reported. Top row in cells represents accuracy in % correct; Bottom row represents reactions times in ms.

Table 4. Null model for the performance in the distractor conditions of WM-/WM+.

|  | **β** | **Std. Error** | | | **95%-CI** | |  |
| --- | --- | --- | --- | --- | --- | --- | --- |
| **intercept** | 73.50 | | 1.28 | 70.98 | | 76.03 | *** |
| **time** | 3.82 | | 1.20 | 1.45 | | 6.19 | ** |
| **sham** | -0.15 | | 1.77 | -3.64 | | 3.33 |  |
| **conventional** | 0.74 | | 1.78 | -2.76 | | 4.25 |  |
| **time x sham** | -0.88 | | 1.66 | -4.16 | | 2.39 |  |
| **time x conventional** | -1.28 | | 1.67 | -4.58 | | 2.01 |  |

*Annotations.* Model: performance ~ time x stimulation + (1|participant). Random intercept SD = 5.99. ***p < .001; **p < .01; *p < .05; p < .1

Table 5. Null model for the performance in the no distractor conditions of WM-/WM+.

|  | **β** | **Std. Error** | | | **95%-CI** | |  |
| --- | --- | --- | --- | --- | --- | --- | --- |
| **intercept** | 80.16 | | 1.07 | 78.06 | | 82.27 | *** |
| **time** | 4.02 | | 1.02 | 2.02 | | 6.03 | *** |
| **sham** | -1.34 | | 1.48 | -4.25 | | 1.57 |  |
| **conventional** | 0.17 | | 1.49 | -2.76 | | 3.09 |  |
| **time x sham** | 0.06 | | 1.40 | -2.71 | | 2.83 |  |
| **time x conventional** | 0.23 | | 1.41 | -2.56 | | 3.02 |  |

*Annotations.* Model: performance ~ time x stimulation + (1|participant). Random intercept SD = 4.94. ***p < .001; **p < .01; *p < .05; p < .1

Table 6. Null model for the overall WM-/WM+ performance.

|  | **β** | **Std. Error** | | | **95%-CI** | |  |
| --- | --- | --- | --- | --- | --- | --- | --- |
| **intercept** | 76.83 | | 1.05 | 74.80 | | 78.87 | *** |
| **time** | 3.93 | | 0.77 | 2.44 | | 5.43 | *** |
| **sham** | -0.74 | | 1.44 | -3.55 | | 2.07 |  |
| **conventional** | 0.47 | | 1.45 | -2.36 | | 3.30 |  |
| **time x sham** | -0.43 | | 1.06 | -2.50 | | 1.63 |  |
| **time x conventional** | -0.55 | | 1.06 | -2.62 | | 1.53 |  |

*Annotations.* Model: performance ~ time x stimulation + (1|participant). Random intercept SD = 5.60. ***p < .001; **p < .01; *p < .05; p < .1

Table 7. Full linear mixed model for the overall WM-/WM+ performance.

|  | **β** | **Std. Error** | **95%-CI** | |  |
| --- | --- | --- | --- | --- | --- |
| **intercept** | 69.35 | 4.99 | 59.52 | 79.18 | *** |
| **time** | -4.16 | 3.82 | -11.70 | 3.38 |  |
| **WMC** | 3.16 | 2.07 | -0.91 | 7.24 |  |
| **sham** | 0.73 | 6.13 | -11.36 | 12.82 |  |
| **conventional** | -4.49 | 6.31 | -16.92 | 7.94 |  |
| **Time x WMC** | 3.42 | 1.58 | 0.29 | 6.54 | * |
| **Time x sham** | 10.90 | 4.69 | 1.63 | 20.17 | * |
| **Time x conventional** | 8.33 | 4.83 | -1.21 | 17.86 | . |
| **WMC x sham** | -0.43 | 2.59 | -5.54 | 4.67 |  |
| **WMC x conventional** | 2.36 | 2.65 | -2.87 | 7.58 |  |
| **Time x WMC x sham** | -4.89 | 1.98 | -8.81 | -0.97 | * |
| **Time x WMC x conventional** | -3.77 | 2.03 | -7.77 | 0.24 | . |

*Annotations.* Model: performance ~ time x stimulation x WM capacity + (1|participant). Random intercept (1|participant) SD = 5.06. ***p < .001; **p < .01; *p < .05; p < .1.

Table 8. Full linear mixed model for the no distractor conditions of WM-/WM+.

|  | **β** | **Std. Error** | **95%-CI** | |  |
| --- | --- | --- | --- | --- | --- |
| **intercept** | 74.24 | 5.30 | 63.81 | 84.67 | *** |
| **time** | -1.94 | 5.23 | -12.26 | 8.39 |  |
| **WMC** | 2.50 | 2.19 | -1.82 | 6.82 |  |
| **sham** | 1.66 | 6.51 | -11.16 | 14.48 |  |
| **conventional** | -4.29 | 6.70 | -17.47 | 8.90 |  |
| **Time x WMC** | 2.52 | 2.17 | -1.76 | 6.80 |  |
| **Time x sham** | 4.69 | 6.43 | -8.01 | 17.38 |  |
| **Time x conventional** | 5.78 | 6.61 | -7.28 | 18.83 |  |
| **WMC x sham** | -1.18 | 2.75 | -6.59 | 4.24 |  |
| **WMC x conventional** | 2.10 | 2.82 | -3.44 | 7.65 |  |
| **Time x WMC x sham** | -1.91 | 2.72 | -7.28 | 3.45 |  |
| **Time x WMC x conventional** | -2.33 | 2.78 | -7.82 | 3.16 |  |

*Annotations.* Model: performance ~ time x stimulation x working memory capacity + (1|participant). ***p < .001; *p < .05; .p < .1

|  | **WM+/WM-** | | | | **WM-** | | | | **WM+** | | | |
| --- | --- | --- | --- | --- | --- | --- | --- | --- | --- | --- | --- | --- |
|  | **β** | **Std. Error** | **95%-CI** |  | **β** | **Std. Error** | **95%-CI** |  | **β** | **Std. Error** | **95%-CI** |  |
| (Intercept) | 70.08 | 3.57 | 63.05 – 77.10 | *** | 75.90 | 3.79 | 68.44 – 83.36 | ******* | 64.23 | 4.45 | 55.47 – 72.99 | ******* |
| time | 6.74 | 2.73 | 1.36 – 12.12 | * | 2.75 | 3.74 | -4.62 – 10.11 |  | 10.80 | 4.33 | 2.27 – 19.33 | * |
| WMC | 2.73 | 1.56 | -0.35 – 5.81 |  | 1.32 | 1.66 | -1.94 – 4.59 |  | 4.13 | 1.95 | 0.30 – 7.97 | * |
| frontoparietal | -0.73 | 6.13 | -12.81 – 11.35 |  | -1.66 | 6.51 | -14.48 – 11.17 |  | 0.22 | 7.65 | -14.85 – 15.29 |  |
| conventional | -5.22 | 5.25 | -15.56 – 5.13 |  | -5.95 | 5.58 | -16.93 – 5.04 |  | -4.54 | 6.55 | -17.45 – 8.37 |  |
| time x WMC | -1.47 | 1.20 | -3.83 – 0.89 |  | 0.61 | 1.64 | -2.62 – 3.83 |  | -3.56 | 1.90 | -7.30 – 0.17 |  |
| time x frontoparietal | -10.90 | 4.69 | -20.15 – -1.66 | * | -4.68 | 6.43 | -17.35 – 7.98 |  | -17.20 | 7.44 | -31.87 – -2.54 | * |
| time x conventional | -2.57 | 4.02 | -10.50 – 5.35 |  | 1.09 | 5.51 | -9.76 – 11.94 |  | -6.23 | 6.38 | -18.80 – 6.34 |  |
| WMC x frontoparietal | 0.43 | 2.59 | -4.67 – 5.54 |  | 1.18 | 2.75 | -4.24 – 6.59 |  | -0.31 | 3.23 | -6.68 – 6.06 |  |
| WMC x conventional | 2.79 | 2.28 | -1.70 – 7.28 |  | 3.28 | 2.42 | -1.49 – 8.05 |  | 2.33 | 2.84 | -3.28 – 7.93 |  |
| time x WMC x frontoparietal | 4.89 | 1.98 | 0.98 – 8.80 | * | 1.91 | 2.72 | -3.44 – 7.26 |  | 7.88 | 3.15 | 1.69 – 14.08 | * |
| time x WMC x  conventional | 1.12 | 1.75 | -2.32 – 4.56 | 0. | -0.42 | 2.39 | -5.13 – 4.29 |  | 2.66 | 2.77 | -2.79 – 8.12 |  |

Table 9. Linear mixed models for conditions of WM+/WM- with sham as reference stimulation group to allow for direct comparisons of sham and conventional stimulation.

*Annotations.* Model: performance ~ time x stimulation x working memory capacity + (1|participant). ***p < .001; *p < .05; .p < .1

Table 10. Correlations between WM capacity and RT time differences across groups.

|  | **Overall** | **No distractor** | **Distractor** |
| --- | --- | --- | --- |
| **sham** | -.09^K^ | -.10 ^K^ | -.07 ^K^ |
| **frontoparietal** | -.02 | -.03 ^K^ | .02 |
| **conventional** | -.19 | -.15 | -.22 |

*Annotations*. Correlations flagged with ^K^ were run with Kendall’s method due to non-normally distributed data. All other correlations were run via Pearson’s method.

Table 11. Linear mixed models for log-transformed reaction times for all conditions of WM-/WM+.

|  |  | **β** | **Std. Error** | **95%-CI** | |  |
| --- | --- | --- | --- | --- | --- | --- |
|  | **overall** |  |  |  | |  |
|  | intercept | 6.87 | 0.02 | 6.83 | 6.91 | *** |
|  | time | -0.06 | 0.01 | -0.09 | -0.04 | *** |
|  |  |  |  |  |  |  |
|  | **No distractor** |  |  |  |  |  |
|  | intercept | 6.84 | 0.02 | 6.80 | 6.88 | *** |
|  | time | -0.07 | 0.01 | -0.09 | -0.04 | *** |
|  |  |  |  |  |  |  |
|  | **distractor** |  |  |  |  |  |
|  | intercept | 6.89 | 0.02 | 6.85 | 6.94 | *** |
|  | time | -0.06 | 0.01 | -0.09 | -0.03 | *** |

*Annotations.* Model: performance ~ time + (1|participant). The dependent variable reaction time was log transformed to ensure normal distribution. ***p < .001; *p < .05; p < .1

Table 12. Linear mixed models for untransformed reaction times for all conditions of WM-/WM+.

|  |  | **β** | **Std. Error** | **95%-CI** | |  |
| --- | --- | --- | --- | --- | --- | --- |
|  | **overall** |  |  |  | |  |
|  | intercept | 986.80 | 21.19 | 945.06 | 1028.53 | *** |
|  | time | -56.37 | 14.18 | -84.30 | -28.44 | *** |
|  |  |  |  |  |  |  |
|  | **No distractor** |  |  |  |  |  |
|  | intercept | 949.80 | 20.55 | 909.33 | 990.33 | *** |
|  | time | -55.87 | 13.59 | -82.64 | -29.11 | *** |
|  |  |  |  |  |  |  |
|  | **distractor** |  |  |  |  |  |
|  | intercept | 1012.79 | 21.66 | 970.13 | 1055.45 | *** |
|  | time | -55.45 | 15.20 | -85.40 | -25.51 | *** |

*Annotations.* Model: performance ~ time + (1|participant). The dependent variable reaction time was log transformed to ensure normal distribution. ***p < .001; *p < .05; p < .1
